# Bis[6-(1*H*-benzimidazol-2-yl-κ*N* ^3^)pyridine-2-carboxyl­ato-κ^2^ *N*,*O*]cobalt(II) dihydrate

**DOI:** 10.1107/S1600536811053700

**Published:** 2011-12-21

**Authors:** Liying Han, Dajun Sun

**Affiliations:** a Department of Gynecology, The Second Hospital of Jilin University, Changchun 130041, People’s Republic of China; b Department of Vascular Surgery, The China-Japan Union Hospital of Jilin University, Changchun 130033, People’s Republic of China

## Abstract

In the title compound, [Co(C_13_H_8_N_3_O_2_)_2_]·2H_2_O, the Co^II^ atom has a distorted octa­hedral environment defined by four N atoms and two O atoms from two 6-(1*H*-benzimidazol-2-yl)pyridine-2-carboxyl­ate ligands. In the crystal, the complex mol­ecules and uncoordinated water mol­ecules are linked *via* N—H⋯O and O—H⋯O hydrogen bonds, forming a two-dimensional supra­molecular structure parallel to (010). π–π inter­actions are present between the imidazole, pyridine and benzene rings [centroid–centroid distances = 3.528 (2), 3.592 (2), 3.680 (2) and 3.732 (3) Å].

## Related literature

For background to supra­molecular architectures, see: Chun *et al.* (2005 ▶); Tranchemontagne *et al.* (2009 ▶). For related complexes with multidentate ligands, see: Eubank *et al.* (2011 ▶); Wang *et al.* (2009 ▶).
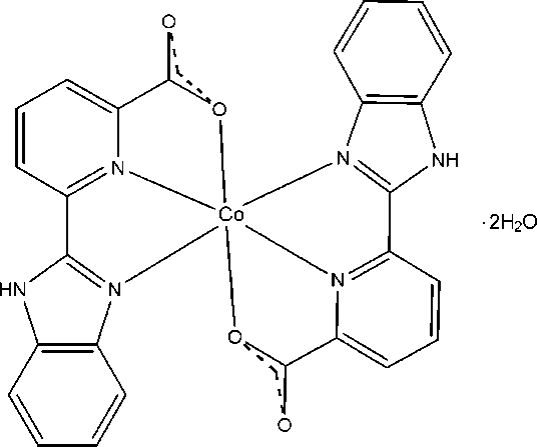


## Experimental

### 

#### Crystal data

[Co(C_13_H_8_N_3_O_2_)_2_]·2H_2_O
*M* *_r_* = 571.41
Monoclinic, 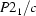

*a* = 9.8602 (5) Å
*b* = 20.3681 (11) Å
*c* = 13.1069 (7) Å
β = 111.453 (1)°
*V* = 2449.9 (2) Å^3^
*Z* = 4
Mo *K*α radiation
μ = 0.76 mm^−1^
*T* = 185 K
0.24 × 0.15 × 0.12 mm

#### Data collection

Bruker APEXII CCD diffractometer
Absorption correction: multi-scan (*SADABS*; Bruker, 2001 ▶) *T*_min_ = 0.839, *T*_max_ = 0.915
13443 measured reflections
4827 independent reflections
3864 reflections with *I* > 2σ(*I*)
*R*_int_ = 0.026

#### Refinement

*R*[*F* ^2^ > 2σ(*F* ^2^)] = 0.049
*wR*(*F* ^2^) = 0.150
*S* = 1.06
4827 reflections
352 parameters
6 restraints
H-atom parameters constrained
Δρ_max_ = 0.96 e Å^−3^
Δρ_min_ = −0.59 e Å^−3^

### 

Data collection: *APEX2* (Bruker, 2007 ▶); cell refinement: *SAINT* (Bruker, 2007 ▶); data reduction: *SAINT*; program(s) used to solve structure: *SHELXTL* (Sheldrick, 2008 ▶); program(s) used to refine structure: *SHELXTL*; molecular graphics: *XP* in *SHELXTL* and *Mercury* (Macrae *et al.*, 2006 ▶); software used to prepare material for publication: *SHELXTL* and *PLATON* (Spek, 2009 ▶).

## Supplementary Material

Crystal structure: contains datablock(s) global, I. DOI: 10.1107/S1600536811053700/hy2495sup1.cif

Structure factors: contains datablock(s) I. DOI: 10.1107/S1600536811053700/hy2495Isup2.hkl

Additional supplementary materials:  crystallographic information; 3D view; checkCIF report

## Figures and Tables

**Table 1:** Hydrogen-bond geometry (Å, °)

*D*—H⋯*A*	*D*—H	H⋯*A*	*D*⋯*A*	*D*—H⋯*A*
N3—H3⋯O3^i^	0.88	2.21	2.917 (4)	137
N6—H6⋯O1^ii^	0.88	2.30	2.971 (4)	133
O1*W*—H1*A*⋯O3^iii^	0.87	2.26	2.923 (4)	134
O1*W*—H1*B*⋯O4	0.86	1.87	2.727 (6)	170
O2*W*—H2*A*⋯O2	0.89	2.21	2.962 (6)	142
O2*W*—H2*B*⋯O1*W*^iii^	0.91	2.05	2.867 (8)	149

Symmetry codes: (i) 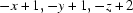; (ii) 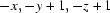; (iii) 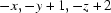.

**Fig. 1.**
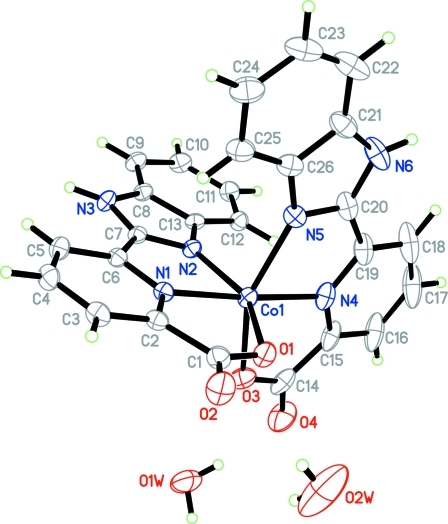
The molecular structure of the title compound. Displacement ellipsoids are drawn at the 30% probability level.

**Fig. 2.**
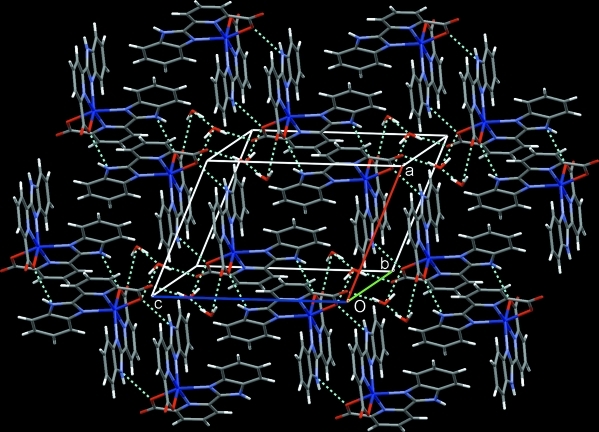
View of the two-dimensional structure of the title compound built by hydrogen bonds (dashed lines).

## supplementary crystallographic information

### Comment

Molecular self-assembly of supramolecular architectures has received much attention during recent decades (Chun *et al.*, 2005; Tranchemontagne *et al.*, 2009). The structures and properties of such systems depend on the coordination and geometric preferences of both the central metal ions and bridging building blocks, as well as on the influence of weaker non-covalent interactions, such as hydrogen bonds and π–π stacking interactions. Multidentate ligands containing rich coordination sites (N and/or O donors) are often employed to produce polymeric networks with structural diversity owing to their various coordination modes (Eubank *et al.*, 2011; Wang *et al.*, 2009). Recently, we obtained the title mononuclear complex by the reaction of cobalt chloride with 6-(1*H*-benzimidazol-2-yl)pyridine-2-carboxylic acid using hydrothermal methods and its crystal structure is reported here.

In the title compound, the Co^II^ atom has a distorted octahedral environment with four N atoms and two carboxylate O atoms from two 6-(1*H*-benzimidazol-2-yl)pyridine-2-carboxylate ligands (Fig. 1). The bond lengths and angles around the Co atom are normal. The complex molecules and uncoordinated water molecules are connected into a two-dimensional structure by extensive N—H···O and O—H···O hydrogen bonds (Fig. 2, Table 1). π–π interactions between the imidazole, pyridine and benzene rings are present [centroid–centroid distances = 3.528 (2), 3.592 (2), 3.680 (2) and 3.732 (3) Å].

### Experimental

The synthesis was performed under hydrothermal conditions. A mixture of CoCl_2_.6H_2_O (0.2 mmol, 0.050 g), 6-(1*H*-benzimidazol-2-yl)pyridine-2-carboxylic acid (0.4 mmol, 0.098 g), NaOH (0.4 mmol, 0.016 g) and H_2_O (15 ml) in a 25 ml stainless steel reactor with a Teflon liner was heated from 293 to 433 K in 2 h and a constant temperature was maintained at 433 K for 72 h. After the mixture was cooled to 298 K, pink crystals of the title compound were recovered from the reaction.

### Refinement

H atoms on C and N atoms were positioned geometrically and refined as riding atoms, with C—H = 0.95 and N—H = 0.88 Å and with *U*_iso_(H) = 1.2*U*_eq_(C, N). H atoms of the water molecules were located from a difference Fourier map and refined as riding, with *U*_iso_(H) = 1.5*U*_eq_(O).

### Crystal data

**Table:**

[Co(C_13_H_8_N_3_O_2_)_2_]·2H_2_O	*F*(000) = 1172
*M**_r_* = 571.41	*D*_x_ = 1.549 Mg m^−^^3^
Monoclinic, *P*2_1_/*c*	Mo *K*α radiation, λ = 0.71073 Å
Hall symbol: -P 2ybc	Cell parameters from 4827 reflections
*a* = 9.8602 (5) Å	θ = 1.0–26.0°
*b* = 20.3681 (11) Å	µ = 0.76 mm^−^^1^
*c* = 13.1069 (7) Å	*T* = 185 K
β = 111.453 (1)°	Block, pink
*V* = 2449.9 (2) Å^3^	0.24 × 0.15 × 0.12 mm
*Z* = 4	

### Data collection

**Table:**

Bruker APEXII CCD diffractometer	4827 independent reflections
Radiation source: fine-focus sealed tube	3864 reflections with *I* > 2σ(*I*)
graphite	*R*_int_ = 0.026
φ and ω scans	θ_max_ = 26.1°, θ_min_ = 2.0°
Absorption correction: multi-scan (*SADABS*; Bruker, 2001)	*h* = −12→10
*T*_min_ = 0.839, *T*_max_ = 0.915	*k* = −23→25
13443 measured reflections	*l* = −16→16

### Refinement

**Table:**

Refinement on *F*^2^	Primary atom site location: structure-invariant direct methods
Least-squares matrix: full	Secondary atom site location: difference Fourier map
*R*[*F*^2^ > 2σ(*F*^2^)] = 0.049	Hydrogen site location: inferred from neighbouring sites
*wR*(*F*^2^) = 0.150	H-atom parameters constrained
*S* = 1.06	*w* = 1/[σ^2^(*F*_o_^2^) + (0.0767*P*)^2^ + 2.8721*P*] where *P* = (*F*_o_^2^ + 2*F*_c_^2^)/3
4827 reflections	(Δ/σ)_max_ = 0.001
352 parameters	Δρ_max_ = 0.96 e Å^−^^3^
6 restraints	Δρ_min_ = −0.59 e Å^−^^3^

### Special details

**Table:**

Geometry. All esds (except the esd in the dihedral angle between two l.s. planes) are estimated using the full covariance matrix. The cell esds are taken into account individually in the estimation of esds in distances, angles and torsion angles; correlations between esds in cell parameters are only used when they are defined by crystal symmetry. An approximate (isotropic) treatment of cell esds is used for estimating esds involving l.s. planes.
Refinement. Refinement of F^2^ against ALL reflections. The weighted R-factor wR and goodness of fit S are based on F^2^, conventional R-factors R are based on F, with F set to zero for negative F^2^. The threshold expression of F^2^ > 2sigma(F^2^) is used only for calculating R-factors(gt) etc. and is not relevant to the choice of reflections for refinement. R-factors based on F^2^ are statistically about twice as large as those based on F, and R- factors based on ALL data will be even larger.

### Fractional atomic coordinates and isotropic or equivalent isotropic displacement parameters (Å^2^)

**Table:**

	*x*	*y*	*z*	*U*_iso_*/*U*_eq_	
C1	0.1555 (4)	0.35179 (16)	0.7579 (3)	0.0386 (8)	
C2	0.3187 (4)	0.35664 (15)	0.8211 (3)	0.0321 (7)	
C3	0.4130 (4)	0.30476 (16)	0.8634 (3)	0.0384 (8)	
H3A	0.3779	0.2609	0.8558	0.046*	
C4	0.5581 (4)	0.31795 (16)	0.9164 (3)	0.0404 (8)	
H4A	0.6245	0.2829	0.9462	0.048*	
C5	0.6094 (4)	0.38247 (16)	0.9270 (3)	0.0356 (7)	
H5A	0.7100	0.3920	0.9631	0.043*	
C6	0.5091 (3)	0.43192 (15)	0.8833 (2)	0.0290 (6)	
C7	0.5339 (3)	0.50287 (14)	0.8836 (2)	0.0278 (6)	
C8	0.6304 (3)	0.60145 (15)	0.9040 (3)	0.0308 (7)	
C9	0.7184 (4)	0.65706 (16)	0.9248 (3)	0.0393 (8)	
H9A	0.8210	0.6545	0.9619	0.047*	
C10	0.6487 (4)	0.71598 (17)	0.8885 (3)	0.0420 (8)	
H10A	0.7052	0.7550	0.9002	0.050*	
C11	0.4972 (4)	0.72036 (16)	0.8348 (3)	0.0388 (8)	
H11A	0.4534	0.7621	0.8127	0.047*	
C12	0.4113 (4)	0.66545 (15)	0.8138 (3)	0.0337 (7)	
H12A	0.3087	0.6684	0.7771	0.040*	
C13	0.4794 (3)	0.60507 (14)	0.8482 (3)	0.0287 (6)	
C14	0.0579 (4)	0.57477 (19)	0.8491 (4)	0.0512 (10)	
C15	0.0334 (4)	0.60199 (19)	0.7370 (3)	0.0502 (10)	
C16	−0.0381 (5)	0.6601 (2)	0.6968 (4)	0.0728 (16)	
H16A	−0.0828	0.6848	0.7375	0.087*	
C17	−0.0424 (6)	0.6812 (2)	0.5950 (4)	0.088 (2)	
H17A	−0.0897	0.7214	0.5657	0.105*	
C18	0.0215 (6)	0.6445 (2)	0.5353 (4)	0.0763 (16)	
H18A	0.0188	0.6586	0.4655	0.092*	
C19	0.0896 (4)	0.58621 (18)	0.5817 (3)	0.0498 (10)	
C20	0.1695 (4)	0.54082 (17)	0.5376 (3)	0.0446 (9)	
C21	0.2695 (5)	0.49105 (19)	0.4359 (3)	0.0518 (10)	
C22	0.3211 (6)	0.4676 (3)	0.3561 (4)	0.0721 (14)	
H22A	0.2964	0.4887	0.2870	0.087*	
C23	0.4077 (6)	0.4137 (3)	0.3816 (4)	0.0718 (14)	
H23A	0.4428	0.3967	0.3283	0.086*	
C24	0.4476 (5)	0.3820 (2)	0.4829 (4)	0.0620 (11)	
H24A	0.5105	0.3450	0.4976	0.074*	
C25	0.3962 (4)	0.40391 (19)	0.5622 (3)	0.0486 (9)	
H25A	0.4216	0.3824	0.6310	0.058*	
C26	0.3061 (4)	0.45864 (17)	0.5370 (3)	0.0409 (8)	
N1	0.3673 (3)	0.41784 (12)	0.8317 (2)	0.0284 (5)	
N2	0.4214 (3)	0.54241 (12)	0.8366 (2)	0.0287 (6)	
N3	0.6612 (3)	0.53553 (13)	0.9257 (2)	0.0334 (6)	
H3	0.7476	0.5182	0.9605	0.040*	
N4	0.0940 (3)	0.56671 (14)	0.6808 (3)	0.0421 (7)	
N5	0.2413 (3)	0.49147 (13)	0.5994 (2)	0.0363 (6)	
N6	0.1837 (4)	0.54260 (16)	0.4400 (3)	0.0599 (10)	
H6	0.1447	0.5718	0.3879	0.072*	
O1	0.0911 (2)	0.40631 (11)	0.7254 (2)	0.0416 (6)	
O2	0.0977 (3)	0.29740 (13)	0.7412 (3)	0.0601 (8)	
O1W	0.1067 (4)	0.5642 (2)	1.1243 (4)	0.1120 (16)	
H1A	0.0385	0.5579	1.1501	0.168*	
H1B	0.0679	0.5744	1.0559	0.168*	
O2W	−0.2043 (5)	0.3346 (3)	0.7130 (6)	0.179 (3)	
H2A	−0.1389	0.3089	0.7008	0.268*	
H2B	−0.1663	0.3533	0.7805	0.268*	
O3	0.1308 (3)	0.52097 (12)	0.8735 (2)	0.0477 (6)	
O4	0.0139 (3)	0.60542 (18)	0.9118 (3)	0.0748 (10)	
Co1	0.22295 (4)	0.49008 (2)	0.75644 (4)	0.03024 (16)	

### Atomic displacement parameters (Å^2^)

**Table:**

	*U*^11^	*U*^22^	*U*^33^	*U*^12^	*U*^13^	*U*^23^
C1	0.0422 (19)	0.0317 (18)	0.0419 (19)	−0.0054 (15)	0.0154 (16)	0.0014 (14)
C2	0.0403 (18)	0.0260 (16)	0.0333 (16)	−0.0007 (13)	0.0174 (14)	0.0001 (12)
C3	0.051 (2)	0.0225 (16)	0.0437 (19)	0.0005 (14)	0.0198 (16)	0.0011 (13)
C4	0.048 (2)	0.0294 (17)	0.0440 (19)	0.0130 (15)	0.0165 (16)	0.0032 (14)
C5	0.0355 (17)	0.0300 (17)	0.0404 (18)	0.0058 (13)	0.0128 (14)	−0.0023 (14)
C6	0.0319 (16)	0.0270 (16)	0.0303 (16)	0.0031 (12)	0.0140 (13)	−0.0006 (12)
C7	0.0294 (16)	0.0278 (15)	0.0289 (16)	0.0022 (12)	0.0140 (13)	−0.0021 (12)
C8	0.0311 (16)	0.0282 (16)	0.0358 (17)	0.0018 (12)	0.0155 (13)	−0.0029 (13)
C9	0.0330 (17)	0.0362 (18)	0.050 (2)	−0.0053 (14)	0.0168 (16)	−0.0043 (15)
C10	0.044 (2)	0.0309 (18)	0.054 (2)	−0.0087 (15)	0.0213 (17)	−0.0022 (15)
C11	0.0444 (19)	0.0281 (17)	0.045 (2)	0.0003 (14)	0.0172 (16)	0.0027 (14)
C12	0.0337 (17)	0.0282 (16)	0.0384 (18)	0.0024 (13)	0.0122 (14)	0.0026 (13)
C13	0.0317 (16)	0.0262 (15)	0.0330 (16)	−0.0021 (12)	0.0176 (13)	−0.0032 (12)
C14	0.0300 (18)	0.049 (2)	0.073 (3)	0.0037 (16)	0.0162 (18)	−0.020 (2)
C15	0.0327 (19)	0.046 (2)	0.056 (2)	0.0075 (16)	−0.0028 (16)	−0.0218 (18)
C16	0.058 (3)	0.059 (3)	0.068 (3)	0.031 (2)	−0.016 (2)	−0.027 (2)
C17	0.095 (4)	0.052 (3)	0.068 (3)	0.041 (3)	−0.027 (3)	−0.019 (2)
C18	0.098 (4)	0.042 (2)	0.049 (2)	0.020 (2)	−0.020 (2)	−0.0059 (19)
C19	0.054 (2)	0.0320 (18)	0.043 (2)	0.0024 (16)	−0.0060 (17)	−0.0085 (15)
C20	0.054 (2)	0.0291 (18)	0.0378 (19)	−0.0060 (16)	0.0019 (16)	−0.0007 (14)
C21	0.069 (3)	0.045 (2)	0.042 (2)	−0.0137 (19)	0.020 (2)	−0.0004 (17)
C22	0.110 (4)	0.069 (3)	0.049 (3)	−0.016 (3)	0.044 (3)	−0.001 (2)
C23	0.093 (4)	0.080 (3)	0.063 (3)	−0.011 (3)	0.054 (3)	−0.008 (3)
C24	0.070 (3)	0.058 (3)	0.072 (3)	−0.001 (2)	0.042 (2)	−0.013 (2)
C25	0.058 (2)	0.047 (2)	0.051 (2)	0.0019 (18)	0.0310 (19)	−0.0018 (17)
C26	0.048 (2)	0.0347 (18)	0.044 (2)	−0.0115 (15)	0.0210 (16)	−0.0069 (15)
N1	0.0339 (14)	0.0228 (12)	0.0306 (13)	0.0019 (10)	0.0143 (11)	0.0002 (10)
N2	0.0276 (13)	0.0253 (13)	0.0342 (14)	0.0013 (10)	0.0124 (11)	−0.0012 (10)
N3	0.0255 (13)	0.0306 (14)	0.0428 (16)	0.0031 (11)	0.0109 (12)	−0.0021 (12)
N4	0.0343 (15)	0.0312 (15)	0.0485 (18)	0.0017 (12)	0.0004 (13)	−0.0095 (13)
N5	0.0421 (16)	0.0287 (14)	0.0378 (16)	−0.0047 (12)	0.0141 (13)	−0.0024 (11)
N6	0.093 (3)	0.0400 (19)	0.0360 (18)	−0.0024 (18)	0.0105 (18)	0.0061 (14)
O1	0.0357 (13)	0.0314 (12)	0.0534 (15)	−0.0017 (10)	0.0111 (11)	0.0030 (11)
O2	0.0529 (17)	0.0332 (14)	0.082 (2)	−0.0138 (12)	0.0099 (15)	0.0031 (13)
O1W	0.063 (2)	0.130 (3)	0.157 (4)	0.002 (2)	0.056 (2)	0.065 (3)
O2W	0.064 (3)	0.174 (6)	0.278 (8)	−0.023 (3)	0.038 (4)	−0.033 (5)
O3	0.0461 (15)	0.0418 (14)	0.0653 (17)	−0.0004 (11)	0.0321 (13)	−0.0061 (12)
O4	0.0603 (19)	0.086 (2)	0.082 (2)	0.0234 (17)	0.0312 (17)	−0.0224 (18)
Co1	0.0299 (3)	0.0230 (2)	0.0377 (3)	0.00136 (16)	0.01228 (19)	−0.00077 (17)

### Geometric parameters (Å, °)

**Table:**

C1—O2	1.228 (4)	C16—H16A	0.9500
C1—O1	1.273 (4)	C17—C18	1.389 (8)
C1—C2	1.520 (5)	C17—H17A	0.9500
C2—N1	1.324 (4)	C18—C19	1.390 (5)
C2—C3	1.383 (5)	C18—H18A	0.9500
C3—C4	1.369 (5)	C19—N4	1.344 (5)
C3—H3A	0.9500	C19—C20	1.463 (6)
C4—C5	1.396 (5)	C20—N5	1.322 (4)
C4—H4A	0.9500	C20—N6	1.337 (5)
C5—C6	1.381 (4)	C21—N6	1.362 (6)
C5—H5A	0.9500	C21—C22	1.403 (6)
C6—N1	1.343 (4)	C21—C26	1.404 (5)
C6—C7	1.466 (4)	C22—C23	1.356 (7)
C7—N2	1.326 (4)	C22—H22A	0.9500
C7—N3	1.347 (4)	C23—C24	1.398 (7)
C8—N3	1.383 (4)	C23—H23A	0.9500
C8—C9	1.392 (4)	C24—C25	1.386 (5)
C8—C13	1.400 (4)	C24—H24A	0.9500
C9—C10	1.379 (5)	C25—C26	1.388 (5)
C9—H9A	0.9500	C25—H25A	0.9500
C10—C11	1.402 (5)	C26—N5	1.381 (4)
C10—H10A	0.9500	N1—Co1	2.034 (2)
C11—C12	1.369 (5)	N2—Co1	2.137 (3)
C11—H11A	0.9500	N3—H3	0.8800
C12—C13	1.395 (4)	N4—Co1	2.028 (3)
C12—H12A	0.9500	N5—Co1	2.132 (3)
C13—N2	1.384 (4)	N6—H6	0.8800
C14—O4	1.231 (5)	O1—Co1	2.093 (2)
C14—O3	1.285 (5)	O1W—H1A	0.8661
C14—C15	1.506 (6)	O1W—H1B	0.8622
C15—N4	1.318 (5)	O2W—H2A	0.8899
C15—C16	1.380 (5)	O2W—H2B	0.9083
C16—C17	1.388 (8)	O3—Co1	2.144 (3)
			
O2—C1—O1	125.8 (3)	N5—C20—C19	118.9 (3)
O2—C1—C2	119.0 (3)	N6—C20—C19	128.3 (3)
O1—C1—C2	115.1 (3)	N6—C21—C22	133.8 (4)
N1—C2—C3	120.9 (3)	N6—C21—C26	105.9 (3)
N1—C2—C1	112.9 (3)	C22—C21—C26	120.3 (4)
C3—C2—C1	126.3 (3)	C23—C22—C21	117.4 (4)
C4—C3—C2	118.6 (3)	C23—C22—H22A	121.3
C4—C3—H3A	120.7	C21—C22—H22A	121.3
C2—C3—H3A	120.7	C22—C23—C24	122.7 (4)
C3—C4—C5	120.5 (3)	C22—C23—H23A	118.6
C3—C4—H4A	119.7	C24—C23—H23A	118.6
C5—C4—H4A	119.7	C25—C24—C23	120.7 (4)
C6—C5—C4	117.8 (3)	C25—C24—H24A	119.6
C6—C5—H5A	121.1	C23—C24—H24A	119.6
C4—C5—H5A	121.1	C24—C25—C26	117.2 (4)
N1—C6—C5	120.6 (3)	C24—C25—H25A	121.4
N1—C6—C7	110.8 (3)	C26—C25—H25A	121.4
C5—C6—C7	128.6 (3)	N5—C26—C25	129.9 (3)
N2—C7—N3	112.7 (3)	N5—C26—C21	108.5 (3)
N2—C7—C6	119.1 (3)	C25—C26—C21	121.6 (4)
N3—C7—C6	128.2 (3)	C2—N1—C6	121.6 (3)
N3—C8—C9	132.5 (3)	C2—N1—Co1	117.9 (2)
N3—C8—C13	105.7 (3)	C6—N1—Co1	120.3 (2)
C9—C8—C13	121.8 (3)	C7—N2—C13	105.5 (3)
C10—C9—C8	116.4 (3)	C7—N2—Co1	112.7 (2)
C10—C9—H9A	121.8	C13—N2—Co1	141.4 (2)
C8—C9—H9A	121.8	C7—N3—C8	107.1 (3)
C9—C10—C11	122.3 (3)	C7—N3—H3	126.5
C9—C10—H10A	118.9	C8—N3—H3	126.5
C11—C10—H10A	118.9	C15—N4—C19	121.2 (3)
C12—C11—C10	121.1 (3)	C15—N4—Co1	118.5 (3)
C12—C11—H11A	119.5	C19—N4—Co1	119.5 (2)
C10—C11—H11A	119.5	C20—N5—C26	105.4 (3)
C11—C12—C13	117.8 (3)	C20—N5—Co1	112.7 (2)
C11—C12—H12A	121.1	C26—N5—Co1	141.9 (2)
C13—C12—H12A	121.1	C20—N6—C21	107.5 (3)
N2—C13—C12	130.3 (3)	C20—N6—H6	126.2
N2—C13—C8	109.1 (3)	C21—N6—H6	126.2
C12—C13—C8	120.6 (3)	C1—O1—Co1	116.6 (2)
O4—C14—O3	124.6 (4)	H1A—O1W—H1B	109.2
O4—C14—C15	119.7 (4)	H2A—O2W—H2B	110.8
O3—C14—C15	115.6 (3)	C14—O3—Co1	114.7 (3)
N4—C15—C16	121.9 (4)	N4—Co1—N1	174.98 (11)
N4—C15—C14	113.6 (3)	N4—Co1—O1	107.37 (10)
C16—C15—C14	124.4 (4)	N1—Co1—O1	77.25 (10)
C15—C16—C17	117.6 (4)	N4—Co1—N5	77.12 (12)
C15—C16—H16A	121.2	N1—Co1—N5	100.74 (10)
C17—C16—H16A	121.2	O1—Co1—N5	95.12 (10)
C16—C17—C18	121.0 (4)	N4—Co1—N2	98.86 (10)
C16—C17—H17A	119.5	N1—Co1—N2	76.70 (10)
C18—C17—H17A	119.5	O1—Co1—N2	153.43 (10)
C17—C18—C19	117.4 (5)	N5—Co1—N2	94.71 (10)
C17—C18—H18A	121.3	N4—Co1—O3	76.70 (12)
C19—C18—H18A	121.3	N1—Co1—O3	105.66 (10)
N4—C19—C18	121.0 (4)	O1—Co1—O3	89.01 (10)
N4—C19—C20	111.3 (3)	N5—Co1—O3	153.54 (11)
C18—C19—C20	127.6 (4)	N2—Co1—O3	93.05 (10)
N5—C20—N6	112.7 (4)		

### Hydrogen-bond geometry (Å, °)

**Table:**

*D*—H···*A*	*D*—H	H···*A*	*D*···*A*	*D*—H···*A*
N3—H3···O3^i^	0.88	2.21	2.917 (4)	137
N6—H6···O1^ii^	0.88	2.30	2.971 (4)	133
O1W—H1A···O3^iii^	0.87	2.26	2.923 (4)	134
O1W—H1B···O4	0.86	1.87	2.727 (6)	170
O2W—H2A···O2	0.89	2.21	2.962 (6)	142
O2W—H2B···O1W^iii^	0.91	2.05	2.867 (8)	149

Symmetry codes: (i) −*x*+1, −*y*+1, −*z*+2; (ii) −*x*, −*y*+1, −*z*+1; (iii) −*x*, −*y*+1, −*z*+2.
